# Breeding of a Multifoliolate Alfalfa Population Using CRISPR/Cas9-Generated Mutants and Evaluation of Agronomic Traits and Nutritive Value

**DOI:** 10.3390/plants15060953

**Published:** 2026-03-19

**Authors:** Yuxin Wang, Yiyu Wang, Jianglei Wang, Lan Mu, Weiliang Kou, Shuifen Huang, Shaoli Zhou, Ming Cai, Jianghua Chen, Haitao Chen

**Affiliations:** 1 College of Grassland Agriculture, Northwest A&F University, Yangling 712100, China; yuxin.wang@nwafu.edu.cn (Y.W.); wangyiyu@nwafu.edu.cn (Y.W.); wangjianglei0611@163.com (J.W.); 2 College of Landscape Architecture and Horticulture, Southwest Forestry University, Kunming 650224, China; mulan1016@163.com; 3 Sanjie Institute of Forage, Yangling 712100, China; 17899315839@163.com; 4 State Key Laboratory of Plant Diversity and Specialty Crops, Xishuangbanna Tropical Botanical Garden, Chinese Academy of Sciences, Kunming 650223, China; hshuifen173154@swfu.edu.cn (S.H.); zhoushaoli@xtbg.ac.cn (S.Z.); 5 Yunnan Academy of Grassland and Animal Science, Kunming 650212, China; mingcaiok@163.com

**Keywords:** alfalfa, CRISPR/Cas9, *MsPALM1*-edited mutant, multifoliolate leaf, nutritive value

## Abstract

Alfalfa (*Medicago sativa* L.) is a major forage legume worldwide. Developing multifoliolate germplasm has been explored as a strategy to improve forage nutritive value and support more efficient forage livestock production. Here, we developed a multifoliolate population, SJ-ML, using CRISPR/Cas9-generated palmate-like pentafoliate mutants as donor parents. Molecular and phenotypic analyses indicated a stable inheritance of the edited alleles and the multifoliolate trait in SJ-ML. SJ-ML was evaluated under solar greenhouse and field conditions, with the recipient cultivar ‘Aohan’ as the greenhouse control and the commercial cultivars ‘Galaxie-Max’ and ‘GN5’ as field controls. SJ-ML showed a greater leaf area and a higher leaf-to-stem ratio, without reductions in yield or plant height. Nutritive analyses indicated that SJ-ML had a higher crude protein content, relative feed value, digestible dry matter, and dry matter intake, while acid detergent fiber, neutral detergent fiber, and lignin were lower than those of the controls. Across regrowth stages, SJ-ML generally maintained a higher nutritive value than controls. These results support SJ-ML as a multifoliolate germplasm resource for improving nutritive value without a trade-off in agronomic traits, with potential relevance for sustainable agriculture through enhanced forage protein value and a reduced reliance on supplemental protein in some ration contexts.

## 1. Introduction

Alfalfa (*Medicago sativa* L.) is one of the most widely grown perennial legume forages and provides a high crude protein yield per unit area, making it an important source of roughage in ruminant production systems [1,2,3]. Beyond supplying a high protein feed, alfalfa contributes to soil and agroecosystem health through its deep root system, symbiotic nitrogen fixation, and perennial growth habit, which together help reduce soil erosion, improve soil structure and lower the need for synthetic nitrogen fertilizers [4,5,6]. With the increasing demand for high-quality forage, improving the nutritive value of alfalfa herbage has become a central objective in many breeding programs [7,8] because higher-quality forage can enhance protein supply per unit of land and reduce the reliance on supplemental protein sources under comparable inputs of water, fertilizer, and land [9]. In routine evaluation, alfalfa forage nutritive value is described by crude protein (CP), neutral detergent fiber (NDF), acid detergent fiber (ADF), lignin, digestibility indices and the leaf-to-stem ratio [10,11,12]. Alfalfa leaves contain higher concentrations of CP and metabolizable nutrients than stems, while stems contain more structural cell wall fiber and are less digestible [13,14,15,16]. Accordingly, increasing the proportion of leaves in the harvested herbage by increasing leaf biomass, leaf area or the leaf-to-stem ratio is considered a practical strategy to enhance the nutritive value of alfalfa [12].

In cultivated alfalfa, the typical compound leaf morphology is trifoliate, with three leaflets per compound leaf unit. However, plants that contain four or more leaflets per compound leaf have also been reported in natural populations and experimental materials, and are referred to as multifoliolate alfalfa [17,18,19]. Previous studies have reported that multifoliolate plants can have a greater specific leaf weight and a higher leaf area and leaf-to-stem ratio than standard trifoliate cultivars, and the canopy-level photosynthetic capacity may also increase [17,19,20,21]. These observations imply that a higher leaflet number per compound leaf may improve forage nutritive value by increasing the contribution of leaf tissue, which is typically richer in protein and lower in fiber than stems [13,14,15]. However, other studies have found that a multifoliolate cultivar does not consistently result in consistent improvements in forage nutritive value [19,21]. These opposite results may be caused by the diversity of the multifoliate percentage in different genotypes, and only genotypes with a high proportion of multifoliolate leaves (>60–70%) have been reported to show measurable advantages in forage nutritive value [19,21]. Therefore, the objective for cultivating high-nutritional multifoliolate alfalfa should express a high proportion of multifoliolate character under the elite germplasm background. However, segregation ratios and differences in the extent of expression indicated that the multifoliolate traits were under complex genetic control [22]. Apart from genetic factors, the plant developmental stage and environmental cues such as the photoperiod also had a greater influence on the proportion of the multifoliolate phenotype [18,21]. Moreover, cultivated alfalfa is an autotetraploid (2n = 4x = 32), self-incompatible, cross-pollinated species with tetrasomic inheritance [23,24]. This genetic complexity makes it difficult to fix favorable alleles and slows genetic gain, especially for traits with unstable expression [25]. As a result, it is difficult to stably transmit the natural multifoliolate traits into an elite germplasm through traditional phenotype-based crossing breeding programs and display the multifoliolate traits at a high level.

With the development of genome editing technologies, CRISPR/Cas9 technology has become a practical tool for efficiently modifying specific genes in crop species [26,27]. It has been applied to improve a range of agronomic and quality traits in major crop species [28,29]. CRISPR/Cas9 also provides a feasible means to circumvent the difficulties of breeding new multifoliolate alfalfa that can stably express the multifoliolate character at a high level. Diploid *Medicago truncatula* is the model plant of the legume species, and the *PALMATE-LIKE PENTAFOLIATA1* (*PALM1*) gene of *Medicago truncatula* encodes a Cys(2)His(2) zinc-finger transcription factor that plays a key role in compound leaf morphogenesis, and the null *palm1* mutants develop palmate-like pentafoliate leaves with five leaflets clustered at the tip [30]. In our previous study, the *palm1*-type multifoliolate mutants were generated by knocking out the *PALM1* orthologs of alfalfa (*MsPALM1*) with CRISPR/Cas9 and the mutated alleles and phenotypes in transgene-free progeny [31]. However, the consequences of increased leaflet numbers in *palm1*-type multifoliolate alfalfa have not been comprehensively evaluated for whole-plant agronomic performance and forage nutritive value.

In this study, we developed a transgene-free multifoliolate alfalfa population, SJ-ML, using the CRISPR/Cas9-generated *palm1*-type multifoliolate mutant as a donor. Under greenhouse conditions, we compared the agronomic traits and nutritive value of SJ-ML with those of its recipient cultivar ‘Aohan’. In field trials, SJ-ML was evaluated together with two widely grown commercial cultivars, ‘Galaxie-Max’ and ‘GN5’, across multiple harvests in a year. We further examined how nutritive value changed during regrowth after cutting. These experiments assessed whether the stable multifoliolate morphology of SJ-ML could improve forage nutritive value without an obvious agronomic trade-off, addressing the practical need to improve forage quality and productivity for sustainable forage systems.

## 2. Results

### 2.1. Development of the Multifoliolate Population SJ-ML

**Figure 1:**
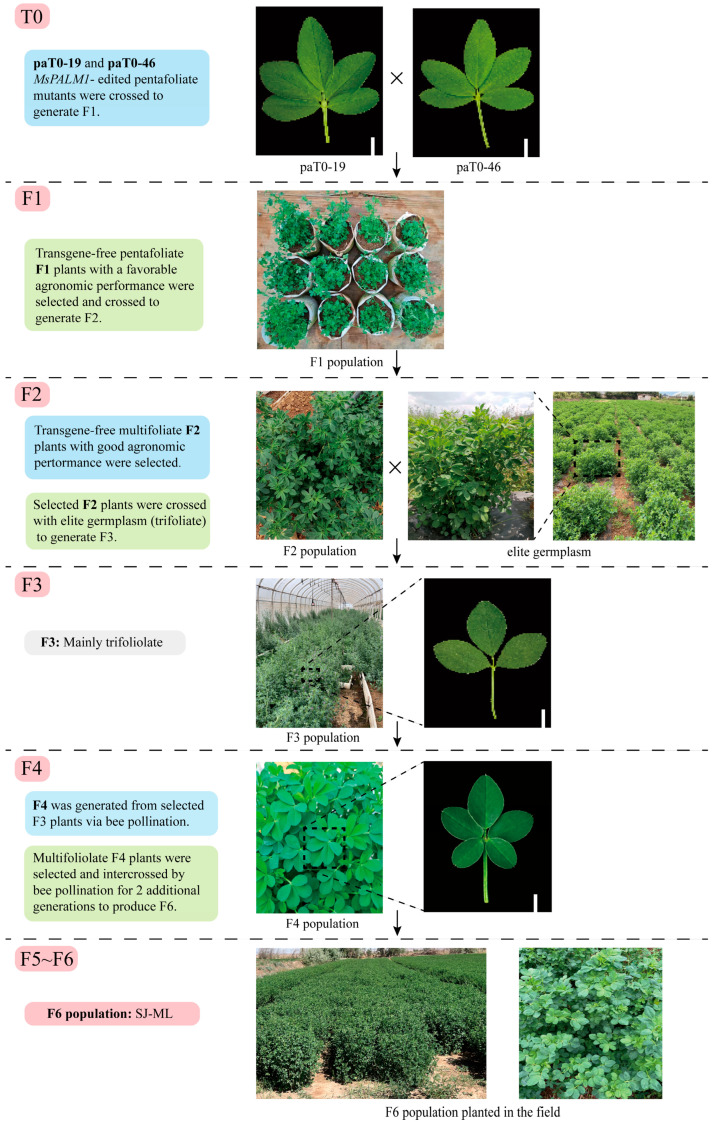
Breeding scheme used to develop the transgene-free multifoliolate alfalfa population SJ-ML. Panels show representative populations and leaf morphologies across generations from T0 to F6. Arrows indicate generation advancement and ‘×’ denotes crossing. The final F6 population was designated SJ-ML. Scale bars = 0.5 cm.

In our previous study, two *MsPALM1*-edited mutants, paT0-19 and paT0-46 [31], with palmate-like pentafoliate leaves were generated using the CRISPR/Cas9 system, with Aohan as the recipient cultivar. Transgene-free F1 progeny with the same palmate-like pentafoliate leaf morphology were subsequently screened from the cross between paT0-19 and paT0-46. To enable subsequent agronomic and nutritive evaluations, transgene-free F1 seedlings bearing palmate-like pentafoliate leaves and four mutated *MsPALM1* alleles were selected as donor parents for cultivating a multifoliolate population in a solar greenhouse breeding program at the Southern Breeding Base of the Sanjie Institute of Forage, Kunming, Yunnan Province, China (Figure 1).

The agronomic traits of the F1 seedlings, including plant height, leaf area, and proportion of multifoliolate leaves, were evaluated in the greenhouse, and individuals with a favorable agronomic performance were manually crossed to generate an F2 population under the same conditions. F2 seedlings with a high multifoliolate proportion were further assessed, and the 20 randomly selected individuals were tested for residual transgene sequences before flowering. No exogenous T-DNA was detected in these 20 F2 individual plants. PCR-restriction enzyme assay (PCR-RE) and Sanger sequencing results showed that all alleles of the *MsPALM1* locus in these plants were mutated and originated from paT0-19 and paT0-46.

Because the field performance of the recipient cultivar ‘Aohan’ was inferior to that of widely grown cultivars, all 20 transgene-free F2 individuals with good agronomic performance were used as donor parents and crossed with elite plants isolated from the field. Individuals from the resulting F3 population were evaluated for agronomic traits, and plants with good traits were selected to generate the F4 population through bee pollination. As unmutated *MsPALM1* alleles derived from the elite field parents were still present, the F3 generation mainly showed trifoliate leaves. Therefore, leaf morphology and the proportion of multifoliolate leaves were first assessed for the F4 population. F4 individuals with palmate-like pentafoliate leaves and a high multifoliolate proportion were selected, followed by comprehensive agronomic evaluation and genotyping of the *MsPALM1* locus using PCR-RE and Sanger sequencing. Multifoliolate F4 plants carrying only mutated *MsPALM1* alleles were then intercrossed through bee-assisted pollination for two additional generations, ultimately resulting in an F6 population designated SJ-ML.

**Figure 2:**
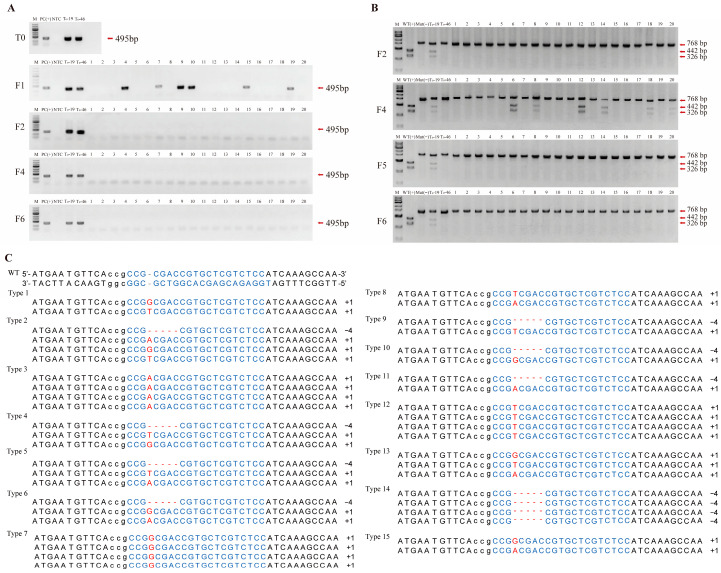
Molecular assessment of residual T-DNA and *MsPALM1* editing across generations. (**A**) PCR was performed to detect residual exogenous T-DNA using primers specific to the *Hpt* gene. Lane order: M, DNA marker; PC(+), plasmid positive control; NTC, no template control; paT0-19; paT0-46; progeny plants (lanes 1–20). (**B**) PCR-restriction enzyme (PCR-RE) assay of the *MsPALM1* locus using BstUI. Lane order: M, DNA marker; WT (digested control), wild-type control; Mut (undigested control), mutant control; paT0-19; paT0-46; progeny plants (lanes 1–20). (**C**) Sanger sequencing of the *MsPALM1* target region in representative F6 plants.

Plants from successive generations (T0 and F1–F6), including the final F6 population SJ-ML, were analyzed using molecular technologies as shown in Figure 2. PCR analysis targeting *Hpt* detected bands in the T0 donor lines paT0-19 and paT0-46 and in a few F1 plants, but no *Hpt* bands were detected in the selected F2–F6 plants (Figure 2A). This indicated that the selected F2–F6 plants were free of detectable residual T-DNA. PCR-RE analysis of *MsPALM1* PCR products showed that most plants from F2 onward displayed the mutant restriction pattern, with only a few F4 plants showing mixed wild-type and mutant fragments; in F5 and F6 plants, the wild-type restriction pattern was absent (Figure 2B). As previously reported, paT0-19 is a chimeric T0 line at the *MsPALM1* target site [31]. Sanger sequencing of *MsPALM1* amplicons from F6 plants confirmed the edited allele combinations at this locus (Figure 2C), indicating that SJ-ML carries only edited *MsPALM1* alleles.

Taken together, these breeding program and molecular analyses show that SJ-ML is a transgene-free, multifoliolate alfalfa population with fixed mutations at the *MsPALM1* locus, providing a stable genetic background for subsequent evaluations of agronomic performance and forage quality.

### 2.2. Greenhouse Agronomic Traits

**Figure 3:**
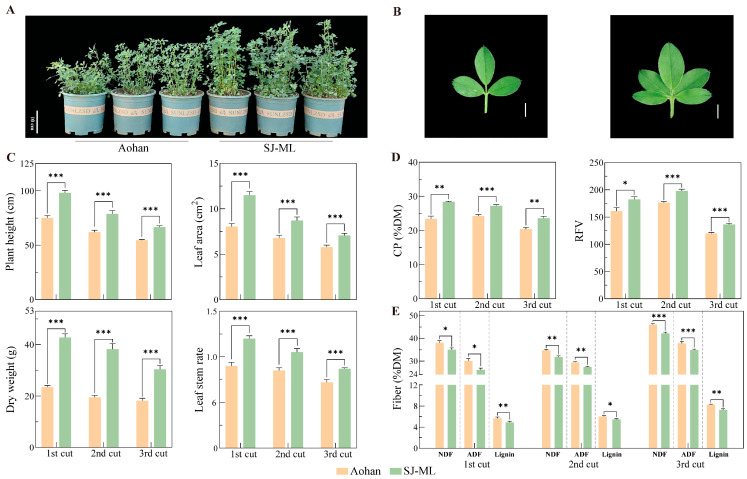
Comparison of SJ-ML and its recipient cultivar ‘Aohan’ under solar greenhouse conditions across three cuts. (**A**) Phenotypes of ‘Aohan’ and SJ-ML grown in pots. Scale bar = 10 cm. (**B**) Representative trifoliolate and palmate-like pentafoliate leaves. Scale bars = 0.5 cm. (**C**) Agronomic traits: plant height, dry weight, leaf area, and leaf-to-stem ratio. (**D**) Nutritive traits: CP and RFV. (**E**) Fiber fractions: NDF, ADF, and lignin; the y-axis is broken between 12 and 24% DM to improve visualization. Bars represent means ± SE. Asterisks (*, **, ***) indicate significant differences between cultivars within the same cut determined using Student’s *t*-test at *p* < 0.05, *p* < 0.01, and *p* < 0.001, respectively.

Alfalfa can be harvested multiple times per year. To assess the stability of agronomic performance, we compared SJ-ML with its recipient cultivar ‘Aohan’ across three consecutive cuts under solar greenhouse conditions (Figure 3; Table S1). Comparisons between SJ-ML and ‘Aohan’ were made within each cut. Visually, SJ-ML showed a more vigorous canopy than ‘Aohan’ (Figure 3A). Consistent with this visual difference, SJ-ML produced significantly greater biomass than ‘Aohan’ across cuts (fresh weight 152.69–251.53 vs. 90.67–137.54 g plant^−1^; dry weight 30.40–42.77 vs. 18.19–23.47 g plant^−1^; Figure 3C; Table S1). When summarized across cuts, SJ-ML showed a significantly higher plant height (+27.0%) and stem diameter (+25.2%) relative to ‘Aohan’ (Figure 3C; Table S1). Compared with ‘Aohan’, in which no multifoliolate leaves were observed (0%), SJ-ML showed a high multifoliolate proportion (76–85%) (Table S1). Associated with this strong multifoliolate expression, SJ-ML also exhibited significantly improved leaf traits, including a larger leaf area and a higher leaf-to-stem ratio (Figure 3C). These results indicate that the multifoliolate population SJ-ML had a clear agronomic advantage over its recipient ‘Aohan’ under greenhouse conditions.

### 2.3. Greenhouse Forage Nutritive Traits

Across three greenhouse cuts during 2025, forage nutritive traits were evaluated on a dry matter basis, with comparisons between SJ-ML and ‘Aohan’ made within each cut (Figure 3; Table S2). SJ-ML showed significantly higher crude protein than ‘Aohan’ across cuts (23.57–28.43% vs. 20.37–24.30% DM), corresponding to a 12.18–21.65% increase relative to ‘Aohan’ in individual cuts (Figure 3D). Higher CP in SJ-ML coincided with reduced fiber fractions: ADF, NDF, and lignin were significantly lower in SJ-ML than in ‘Aohan’ (Figure 3E). These compositional differences translated into higher feeding-value indices, and digestible dry matter (DDM), dry matter intake (DMI), and relative feed value (RFV) were significantly higher in SJ-ML across cuts (Figure 3D; Table S2). By contrast, dry matter (DM) concentration and ether extract (EE) did not differ between cultivars (Table S2). Collectively, SJ-ML had a higher CP and lower fiber fractions than ‘Aohan’, and RFV, DDM, and DMI were correspondingly higher.

### 2.4. Field Agronomic Traits

Following the greenhouse assessment against the recipient cultivar ‘Aohan’, SJ-ML was further evaluated under field conditions to compare its agronomic performance with widely grown commercial cultivars. Because ‘Aohan’ is a Chinese landrace and typically shows a lower yield and forage quality than elite cultivars under standard field management, SJ-ML was compared with the commercial cultivars ‘Galaxie-Max’ and ‘GN5’ across four cuts under standard management.

**Figure 4:**
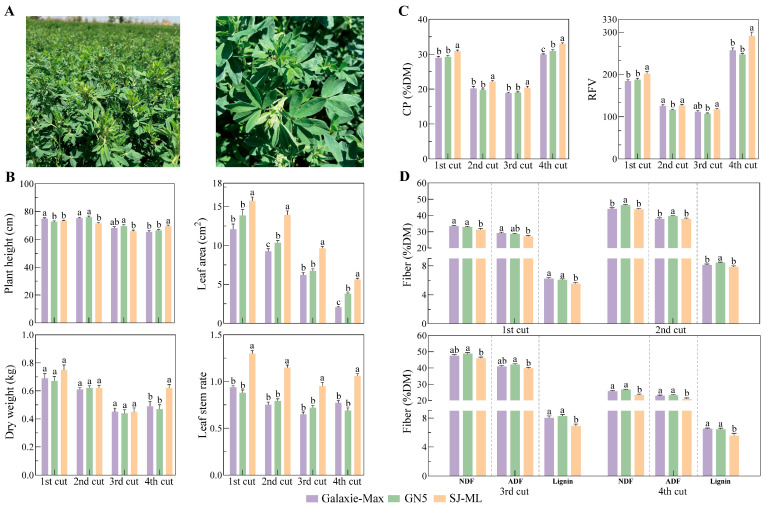
Comparison of SJ-ML and two commercial cultivars ‘Galaxie-Max’ and ‘GN5’ under field conditions across four cuts. (**A**) Field phenotypes of SJ-ML. (**B**) Agronomic traits: plant height, dry weight, leaf area, and leaf-to-stem ratio. (**C**) Nutritive traits: CP and RFV. (**D**) Fiber fractions: NDF, ADF, and lignin; the y-axis is broken between 9 and 20% DM to improve visualization. Bars represent means ± SE. Different lowercase letters above bars indicate significant differences among cultivars within the same cut based on one-way ANOVA followed by Duncan’s multiple range test (DMRT) at *p* < 0.05.

The field agronomic performance of SJ-ML was evaluated across four cuts in 2024–2025, with cultivar comparisons made within each cut (Figure 4B; Table S3). In terms of aboveground biomass, fresh and dry herbage yields of SJ-ML were generally comparable to the controls across all cuts, with minor cut-specific differences. Plant height was also generally similar to the controls, whereas stem diameter in SJ-ML was comparable to or greater than the controls. In contrast to these largely comparable yield and growth traits, SJ-ML showed a high multifoliolate proportion across cuts (50–75%), whereas both control cultivars remained trifoliolate (0% multifoliolate leaves; Table S3). In line with this multifoliolate expression, SJ-ML exhibited a significantly larger leaf area and a higher leaf-to-stem ratio than both ‘Galaxie-Max’ and ‘GN5’ in all cuts (Figure 4B; Table S3).

Overall, SJ-ML showed an agronomic performance comparable to that of the commercial cultivars, while consistently exhibiting a larger leaf area and higher leaf-to-stem ratio under field conditions. Detailed means and significance levels for all traits are shown in Table S3.

### 2.5. Field Forage Nutritive Traits

To compare the forage nutritive value of SJ-ML with that of commercial cultivars, hay samples collected from each of the four cuts were analyzed, and cultivar comparisons were made within each cut (Figure 4C,D; Table S4). DM concentration did not differ among SJ-ML, ‘Galaxie-Max’, and ‘GN5’ in any cut. EE was also generally comparable among cultivars, although SJ-ML was higher than both controls in Cut 4 (Table S4).

Across the four cuts, SJ-ML showed a significantly higher CP than both ‘Galaxie-Max’ and ‘GN5’ in each cut (Figure 4C; Table S4). Overall, CP in SJ-ML ranged from 20.41 to 33.01% DM, corresponding to a 5.64% to 12.54% increase relative to the two commercial controls across cuts (Table S4). Fiber fractions in SJ-ML were also reduced overall (ADF 20.93–39.80% DM; NDF 23.30–46.00% DM; lignin 5.56–7.87% DM; Figure 4D; Table S4), but the cut-specific significance patterns differed by comparator cultivar. Relative to ‘Galaxie-Max’, SJ-ML had a lower ADF and NDF in Cuts 1 and 4 but did not differ in Cuts 2 and 3. Relative to ‘GN5’, SJ-ML had a significantly lower ADF in Cuts 2–4, while NDF was significantly lower in all cuts. Lignin in SJ-ML was significantly lower than both cultivars in Cuts 1, 3, and 4; in Cut 2, lignin was significantly lower than ‘GN5’ but similar to ‘Galaxie-Max’.

Based on ADF and NDF, DDM, DMI, and RFV were calculated (Table S4). Overall, these indices were higher in SJ-ML, showing a more consistent advantage relative to ‘GN5’ and cut-dependent differences relative to ‘Galaxie-Max’; this pattern mirrored the cut-specific differences observed for ADF and NDF (Table S4). RFV ranged from 117.17 to 291.00 in SJ-ML, compared with 111.67–257.33 in ‘Galaxie-Max’ and 106.83–247.17 in ‘GN5’ (Table S4). SJ-ML had a higher RFV than ‘GN5’ in all cuts. Relative to ‘Galaxie-Max’, RFV in SJ-ML was higher in the first and fourth cuts, but similar in the second and third cuts (Figure 4C, Table S4).

Across the four cuts, SJ-ML showed a higher CP and generally lower fiber fractions than the commercial cultivars, with correspondingly higher feeding-value indices (DDM, DMI, and RFV). The differences among cuts are summarized in Table S4.

### 2.6. Dynamics of Forage Nutritive Value During Regrowth

**Figure 5:**
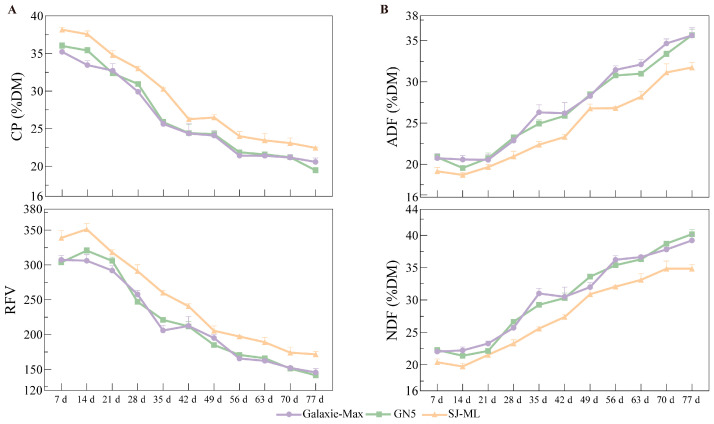
Dynamics of forage nutritive value of SJ-ML and two commercial controls during regrowth under field conditions. SJ-ML, ‘Galaxie-Max’, and ‘GN5’ were sampled at 7-day intervals from early regrowth to the podding stage (days post-harvest). (**A**) Nutritive traits: CP and RFV. (**B**) Fiber fractions: ADF and NDF. Data are shown as mean ± SE.

SJ-ML was compared with ‘Galaxie-Max’ and ‘GN5’ at 7-day intervals from early regrowth to the podding stage in 2025. This analysis revealed clear temporal trends in forage nutritive value among SJ-ML and the two commercial cultivars (Figure 5; Table S5). Across all three entries, the nutritive value declined as regrowth progressed: CP and RFV decreased (Figure 5A), whereas ADF and NDF increased (Figure 5B). Additional traits and derived indices are summarized in Table S5.

From 7 d to 77 d, CP in SJ-ML decreased by 41.2%, whereas ADF and NDF increased by 65.9% and 70.9%, respectively, and RFV decreased by 49.3% (Figure 5A,B). Throughout the regrowth period, SJ-ML generally had a higher CP and feeding-value indices and lower fiber fractions than ‘Galaxie-Max’ and ‘GN5’, as shown in Figure 5 and Table S5, indicating that its higher forage nutritive value was maintained over the entire regrowth period rather than being restricted to a single cutting date.

## 3. Discussion

### 3.1. SJ-ML Is a Stable Multifoliolate Alfalfa Resource

Multifoliolate alfalfa has often been proposed as a way to improve forage quality, because leaves are the main photosynthetic and nutrient storage organs and have a higher crude protein and lower fiber than stems [13,32]. However, the autotetraploid and outcrossing characteristics of cultivated alfalfa make it difficult to fix the natural multifoliolate trait at a high and stable frequency in elite backgrounds using conventional crossing and selection [23,24]. SJ-ML is a transgene-free multifoliolate alfalfa population developed using *MsPALM1*-edited mutants as donors and an elite trifoliolate background, in which the *palm1*-type multifoliolate phenotype is stably inherited. In earlier reports, multifoliolate materials often showed variable genetic backgrounds and unstable or low-frequency expression of the multifoliolate phenotype, which can complicate the interpretation of agronomic and nutritive effects [11,19]. This SJ-ML population provides a useful system to examine how a genetically fixed multifoliolate leaf type performs in terms of agronomic traits and forage nutritive value across environments and during regrowth.

### 3.2. Nutritive Advantages of SJ-ML Relative to the Controls

CP is one of the primary indicators of alfalfa nutritive value because it directly reflects the protein supply to ruminants and is commonly considered in ration formulation [33,34]. In our comparisons, SJ-ML had a higher CP than the landrace cultivar ‘Aohan’ and the commercial cultivars ‘Galaxie-Max’ and ‘GN5’ within the same cut. The reported CP concentrations of alfalfa herbage are often around 15 to 22% DM under typical harvest management [35], and CP can vary substantially with harvest stage and management intensity [36,37]. However, some samples in this study showed CP values above commonly reported ranges. This phenomenon may be caused by the sampling procedures, as all the samples were manually handled under controlled greenhouse or laboratory conditions. Quality evaluation guidelines such as those of the American Forage and Grassland Council (AFGC) emphasize ADF and NDF and use them to derive DDM, DMI, and RFV [38,39]. RFV is a widely used forage quality index [40]. In general, a lower ADF and NDF indicate a greater digestibility and higher intake potential, and lignin is negatively associated with fiber degradability in alfalfa [41,42,43]. Under greenhouse conditions, the NDF, ADF, and lignin of SJ-ML were consistently lower than those in ‘Aohan’ in the same cut. In the field, these fiber fractions in SJ-ML were generally lower than those in ‘GN5’ and were lower than or similar to those in ‘Galaxie-Max’, depending on the cut. Consistent with these fiber patterns, the derived indices (DDM, DMI, and RFV) were higher in SJ-ML than in ‘Aohan’ under greenhouse conditions and were higher than those of at least one of the two commercial cultivars in the field. A greater leaf proportion may partly account for the higher CP concentration and lower fiber fractions in SJ-ML. Because leaves typically contain more protein and less structural fiber than stems, a greater leaf contribution provides a simple structural explanation for the improved nutritive profile of harvested herbage [13,14].

### 3.3. SJ-ML Maintains a Nutritive Advantage Throughout Regrowth

The harvest stage is one of the main factors affecting both yield and forage nutritive value in alfalfa. In our regrowth experiment, SJ-ML and the two commercial cultivars followed the typical pattern for alfalfa stands: as regrowth advanced from early regrowth to the podding stage, CP declined, NDF, ADF, and lignin increased, and DDM, DMI, and RFV decreased [15]. This agrees with previous reports showing that, as alfalfa goes into developmental stages, stem proportion and cell wall lignification increase and stem digestibility declines more rapidly than that of leaves, so that the decline in the whole-plant nutritive value largely reflects a greater contribution of stem tissue to harvested herbage [13,32]. Against this general background, SJ-ML maintained a higher CP and lower fiber fractions than ‘Galaxie-Max’ and ‘GN5’ at most sampling dates from early regrowth to podding, and consequently often had a higher RFV, DDM and DMI. These results suggest that the multifoliolate leaf phenotype of SJ-ML is associated with an improved forage nutritive value across a wide range of regrowth stages, rather than providing an advantage only at a single harvest date. More broadly, the reported quality gains from multifoliolate materials are not always consistent across studies, and little or no improvement has been reported under some genetic backgrounds or management conditions [19,21], which may reflect a variation in the expression level of the multifoliolate phenotype and the genetic background, together with maturity and other developmental traits that jointly influence both the yield and forage nutritive value [11,19,43].

### 3.4. No Obvious Agronomic Trade-Off Associated with the Multifoliolate Trait in SJ-ML

Because *PALM1*/*MsPALM1* encodes a transcription factor [30,31], a common concern is that loss-of-function mutations might have unintended pleiotropic effects on growth or other agronomic traits. In our greenhouse experiment, however, SJ-ML performed better than its recipient cultivar ‘Aohan’ for all traits measured, suggesting that increasing the leaflet number in this genetic background did not impair vegetative growth. Under field conditions, the herbage yield, plant height, and stem diameter of SJ-ML across four cuts were generally comparable to those of the two control cultivars ‘Galaxie-Max’ and ‘GN5’, while the leaf area and leaf-to-stem ratio were consistently higher in SJ-ML than that in the field controls. These observations indicate that a loss-of-function mutation of *PALM1*/*MsPALM1* will not have adverse effects on other agronomic traits, consistent with reports that the multifoliolate phenotype is not necessarily associated with a reduced herbage yield in alfalfa [19]. However, a reduced yield has been reported in some early multifoliolate lines, often attributed to the reduced vigor associated with mild inbreeding during population development and unfavorable genetic backgrounds [21,44], suggesting that apparent yield penalties in multifoliolate materials may reflect population development history and background effects rather than the multifoliolate leaf trait itself.

### 3.5. Breeding Implications, Limitations, and Future Perspectives

In conclusion, the SJ-ML population combined the multifoliolate trait from CRISPR/Cas9-generated mutants with an improved agronomic background through crossing and selection, resulting in a high frequency of the multifoliolate phenotype. Across greenhouse and field assessments, this strong and stable multifoliolate phenotype was generally associated with a higher CP, lower fiber fractions (NDF, ADF, and lignin), and a higher DDM, DMI, and RFV, without a trade-off in agronomic traits. These findings suggest that multifoliolate alfalfa is most likely to show a nutritional advantage when the phenotype is strongly displayed in a genetic background that maintains adequate biomass production, and they provide additional evidence that may help explain why the reported relationships between the multifoliolate phenotype and forage quality improvement have been inconsistent across studies. Notably, higher-quality forage may help increase the protein supply per unit land area under comparable inputs of water, fertilizer, and land, and may reduce the reliance on supplemental protein sources in some production systems [45]. Such changes are often discussed as routes to improved feed efficiency and reduced feed demand per unit of animal products, because the higher digestibility and intake potential can support animal performance with less feed required per unit of output [46,47]. In addition, as a perennial legume, alfalfa contributes to sustainability beyond the harvested forage through biological nitrogen inputs via symbiotic fixation [48]. Together, these considerations link the breeding value of the alfalfa germplasm with an improved nutritive value to broader goals in sustainable agriculture.

The present study suggests SJ-ML as a multifoliolate alfalfa population with potential value as a germplasm for improving nutritive value. Our evaluation, however, was limited to one genetic background and one selection scheme, and was carried out at a small number of sites over a relatively short period, without direct measurements of animal performance. Future work should test SJ-ML and related multifoliolate lines across multiple environments and years, assess traits such as stress tolerance, stand persistence and seed yield, and include feeding trials with ruminants to quantify animal responses to the forage and to support subsequent evaluations of nitrogen and carbon outcomes at the system level. Beyond direct testing, SJ-ML could also be used as a parent in crosses with the elite germplasm differing in fall dormancy score, with the aim of developing multifoliolate lines adapted to different production regions and management systems. Such studies will help clarify how reliably multifoliolate alfalfa derived from *MsPALM1* editing can be used in practical breeding and under different alfalfa dormancy.

## 4. Materials and Methods

### 4.1. Plant Materials and Molecular Characterization

PaT0-19 and paT0-46, two *MsPALM1*-edited multifoliolate mutants in the ‘Aohan’ background, served as donor parents. Using these donor parents, we developed a transgene-free multifoliolate alfalfa population, SJ-ML, through crossing and selection, which was subsequently evaluated in greenhouse and field trials. The recipient cultivar ‘Aohan’ was used as the greenhouse control, and the widely grown commercial cultivars ‘Galaxie-Max’ and ‘GN5’ were used as field controls. Seeds of ‘Aohan’, ‘Galaxie-Max’, and ‘GN5’ were obtained from the Forage Germplasm Repository, Northwest A&F University.

To confirm the transgene-free status during population development, genomic DNA was extracted from plants across different generations (T0 and F1–F6) and tested with PCR using primers *Hpt*-F/*Hpt*-R targeting the Hygromycin phosphotransferase (*Hpt*) gene within the T-DNA region of the vector (primer sequences are shown in Table 1). Each 20 µL PCR reaction contained 10 µL of 2× Magic Taq Super Green Mix (Tolo Biotech Co., Ltd., Shanghai, China), 0.5 µL of each primer (10 µM), 1.0 µL of genomic DNA (~25 ng), and 8.0 µL of nuclease-free water. The PCR program consisted of an initial denaturation at 95 °C for 5 min; 32 cycles of 95 °C for 30 s, 58 °C for 30 s, and 72 °C for 45 s; and a final extension at 72 °C for 7 min. PCR products were separated through 1.5% agarose gel electrophoresis and visualized to determine whether the expected ~495 bp *Hpt* amplicon was detected, and tested plants showing no band were considered free of residual T-DNA.

For *MsPALM1* genotyping, the target region was amplified with primers PA-F/PA-R from the genomic DNA of plants from T0 to F6 generations using the same reaction mix and cycling conditions described above. Amplicons (~768 bp) were gel-purified and primarily analyzed with a PCR-restriction enzyme (PCR-RE) assay using BstUI (New England Biolabs, Ipswich, MA, USA). Amplicons carrying the wild-type allele contain an intact BstUI recognition site at the CRISPR/Cas9 target and can be digested into two fragments (442 and 326 bp). In edited plants, mutations at the target site disrupt this recognition site, yielding an undigested ~768 bp fragment or a mixed restriction pattern. Restriction patterns were visualized on agarose gels to assess the *MsPALM1* editing status. For sequence validation, PCR products from selected plants were cloned into the T-vector pMD™19 (Takara Biomedical Technology (Beijing) Co., Ltd., Beijing, China), and 20 colonies per amplicon were randomly selected for Sanger sequencing.

### 4.2. Greenhouse Experiment

SJ-ML and its recipient cultivar ‘Aohan’ were evaluated in a solar greenhouse at the Southern Breeding Base of the Sanjie Institute of Forage, Kunming, Yunnan, China (102°54′ E, 25°46′ N; 2069 m a.s.l.). Plants were grown in pots filled with a field soil:vermiculite mixture (2:1, *v*/*v*) and managed uniformly. Thirty potted plants per entry were used. Seeds were sown in April 2025, and three cuts were taken during the growing season at regular intervals. Agronomic traits and forage nutritive parameters were assessed at each cut.

For agronomic measurements, all 30 plants per cultivar/population were evaluated immediately before each cut. Plant height was measured from the soil surface to the tip of the tallest stem using a ruler (0.1 cm). Stem diameter was measured at 3 cm above the soil surface using a vernier caliper (0.01 mm). Leaf area was measured with a leaf area meter YMJ-CHA3 (Zhejiang Top Cloud-Agri Technology Co., Ltd., Hangzhou, China) using the third fully expanded compound leaf from the upper canopy, and the proportion of multifoliolate leaves was calculated. For yield traits, plants were cut to a 5 cm stubble height and the fresh weight per plant was recorded immediately. Leaves and stems were then separated and dried at 65 °C for approximately 48 h to a constant weight to measure the dry weight per plant, leaf dry weight, and stem dry weight. The leaf-to-stem ratio was calculated as the leaf dry weight divided by the stem dry weight.

In order to analyze SJ-ML forage nutrition, samples were collected at each cut. For each cultivar/population and cut, six plants were randomly selected and mixed, dried at 65 °C to a constant weight, and ground to pass through a 1 mm sieve. DM, CP, NDF, ADF, lignin, and EE were determined through near-infrared reflectance spectroscopy (NIRS) using an FOSS DS2500F spectrometer (FOSS, Hillerød, Denmark), with spectra collected over 1100–2500 nm (32 scans per sample; 2 nm wavelength intervals). NIRS, a rapid method widely used for routine forage analysis, was applied with models calibrated and validated against reference chemical composition data [49]. Values are expressed on a dry matter basis (% DM). DDM, DMI, and RFV were calculated from ADF and NDF using standard equations [50]:

DDM (% DM) = 88.9 − 0.779 × ADF (% DM),

DMI (% BW) = 120/NDF (% DM),

RFV = (DDM × DMI)/1.29.

### 4.3. Field Experiments

Field experiments were conducted from 2024 to 2025 in the experimental farm at the same site as the greenhouse trial. SJ-ML was compared with two widely grown commercial alfalfa cultivars, ‘Galaxie-Max’ and ‘GN5’. The experiment followed a randomized complete block design with three replicates. Each plot measured 3 m × 5 m (15 m^2^), with 0.5 m alleys between plots. The soil at the site was red soil (pH 6.5).

Seeds were sown on 22 April 2024 after conventional seedbed preparation. Superphosphate was applied as a basal fertilizer at 750 kg ha^−1^. Seeds were hand sown in rows spaced 30 cm apart at a depth of 1–2 cm, at a seeding rate of 30 kg ha^−1^, and the seedbed was rolled after sowing. Plots were managed uniformly, with weed check and irrigation applied as needed; no additional fertilizer was applied during the experimental period.

Four cuts were used for trait evaluation (September 2024 and May, June, and July 2025). At each cut, alfalfa was harvested to a stubble height of 5 cm. For each cultivar/population at each cut, 100 shoots were randomly sampled across the three replicate plots (approximately 33 shoots per plot). Plant height, stem diameter, leaf area, and the proportion of multifoliolate leaves were measured as described for the greenhouse experiment. For yield traits and the leaf-to-stem ratio, two 1 m^2^ quadrats were harvested from each plot (six quadrats per cultivar/population across three plots). Fresh weight was recorded immediately after harvest. Each quadrat was divided into five parts, and the harvested biomass from each part was separated into leaves and stems, which were dried to a constant weight to determine the dry weight and to calculate the leaf-to-stem ratio.

In order to analyze alfalfa nutrition, samples were collected at each cut. For each cultivar/population and cut, six samples (two per plot) were prepared as described for the greenhouse experiment. CP, NDF, ADF, lignin, and EE were determined through NIRS using the same procedure as in the greenhouse experiment, and DDM, DMI, and RFV were calculated from NDF and ADF using the equations described in Section 4.2.

To characterize changes in nutritive value during regrowth, plants were sampled at regular intervals in 2025. After the late July harvest, SJ-ML, ‘Galaxie-Max’, and ‘GN5’ were sampled at approximately 7-day intervals from early regrowth to the podding stage, yielding 11 sampling dates between 4 August and 13 October. On each date, forage was collected from each plot and processed as described above.

### 4.4. Statistical Analysis

All statistical data were analyzed using IBM SPSS Statistics (version 26.0, IBM Corp., Armonk, NY, USA). Greenhouse data were compared between SJ-ML and ‘Aohan’ using Student’s *t*-test within each cut. Field data were analyzed using one-way ANOVA within each cut, followed by Duncan’s multiple range test (DMRT) when significant (*p* < 0.05). Data are presented as mean ± SE.

## 5. Conclusions

In this study, the multifoliolate alfalfa population SJ-ML was generated through crossing and selection using *MsPALM1*-edited multifoliolate mutants as donors, and its agronomic performance and nutritive value were evaluated under greenhouse and field conditions. Compared with the trifoliolate control cultivars, SJ-ML generally maintained a higher nutritional value across the harvest period, specifically manifested as higher crude protein and lower fiber fractions (NDF, ADF, and lignin) with an improved DDM, DMI, and RFV, while agronomic traits were not negatively affected. Together, these results provide a field assessment of *MsPALM1*-edited, genetically stable multifoliolate alfalfa and support its potential as a germplasm for breeding lines that combine improved nutritive value with stable yield performance; under comparable inputs of land, water, and fertilizer, such improvements may increase the protein value obtained from forage and reduce the reliance on supplemental protein sources in some ration formulations, thereby supporting goals in sustainable agriculture. Further multi-year, multi-site trials and feeding studies are needed to test the stability and practical utility of this germplasm under a broader range of production conditions.

## 6. Patents

The method of obtaining multifoliolate *Medicago sativa* by means of *MsPALM1* artificial site-directed mutants has been granted a Chinese patent application (patent number: CN 201810724563.3), United States patent application (patent number: US 11,365,423 B2) and Australian patent application (patent number: 2019297209) by Guangdong Sanjie Forage Biotechnology Co., Ltd. Author H.C. is an advisor to this company.

## Figures and Tables

**Table 1:** The primer sequences used in this study.

Usage	Primer Name	Primer Sequence (5′–3′)	
		Forward	Reverse
Hygromycin detection	*Hpt*	GCATCGGCCGCGCTCCCGAT	GATGTTGGCGACCTCGTATT
Target sequencing	PA	ATGGCTACAGATATTGGCTTTCTTTC	TCAAGTTGGTGTTGGCTTGTTC

## Data Availability

All data supporting the findings of this study are available within the paper and within its Supplementary Materials published online. Further inquiries can be directed to the corresponding author.

## Supplementary Materials

The following supporting information can be downloaded at: https://www.mdpi.com/article/10.3390/plants15060953/s1, Table S1: Greenhouse agronomic traits; Table S2: Greenhouse nutritive traits; Table S3: Field agronomic traits; Table S4: Field nutritive traits; Table S5: Regrowth dynamics of nutritive traits.

## Abbreviations

The following abbreviations are used in this manuscript:

ADF	Acid detergent fiber
BW	Body weight
CP	Crude protein
DDM	Digestible dry matter
DM	Dry matter
DMI	Dry matter intake
DMRT	Duncan’s multiple range test
EE	Ether extract
GN5	Gannong No.5 alfalfa
NDF	Neutral detergent fiber
RFV	Relative feed value
